# Comparative transcriptome combined with morpho‐physiological analyses revealed key factors for differential cadmium accumulation in two contrasting sweet sorghum genotypes

**DOI:** 10.1111/pbi.12795

**Published:** 2017-08-03

**Authors:** Juanjuan Feng, Weitao Jia, Sulian Lv, Hexigeduleng Bao, Fangfang Miao, Xuan Zhang, Jinhui Wang, Jihong Li, Dongsheng Li, Cheng Zhu, Shizhong Li, Yinxin Li

**Affiliations:** ^1^ Key Laboratory of Plant Molecular Physiology Institute of Botany Chinese Academy of Sciences Beijing China; ^2^ College of Life Sciences China Jiliang University Hangzhou China; ^3^ Institute of Nuclear and New Energy Technology Tsinghua University Beijing China; ^4^ Beijing Engineering Research Center for Biofuels Tsinghua University Beijing China

**Keywords:** cadmium, cell wall, endodermis, sweet sorghum, transcriptome, transporter

## Abstract

Cadmium (Cd) is a widespread soil contaminant threatening human health. As an ideal energy plant, sweet sorghum (*Sorghum bicolor* (L.) Moench) has great potential in phytoremediation of Cd‐polluted soils, although the molecular mechanisms are largely unknown. In this study, key factors responsible for differential Cd accumulation between two contrasting sweet sorghum genotypes (high‐Cd accumulation one H18, and low‐Cd accumulation one L69) were investigated. H18 exhibited a much higher ability of Cd uptake and translocation than L69. Furthermore, Cd uptake through symplasmic pathway and Cd concentrations in xylem sap were both higher in H18 than those in L69. Root anatomy observation found the endodermal apoplasmic barriers were much stronger in L69, which may restrict the Cd loading into xylem. The molecular mechanisms underlying these morpho‐physiological traits were further dissected by comparative transcriptome analysis. Many genes involved in cell wall modification and heavy metal transport were found to be Cd‐responsive DEGs and/or DEGs between these two genotypes. KEGG pathway analysis found phenylpropanoid biosynthesis pathway was over‐represented, indicating this pathway may play important roles in differential Cd accumulation between two genotypes. Based on these results, a schematic representation of main processes involved in differential Cd uptake and translocation in H18 and L69 is proposed, which suggests that higher Cd accumulation in H18 depends on a multilevel coordination of efficient Cd uptake and transport, including efficient root uptake and xylem loading, less root cell wall binding, and weaker endodermal apoplasmic barriers.

## Introduction

As global industrialization proceeds, cadmium (Cd) has become one of the most harmful and widespread pollutants in environment (He *et al*., 2013; Xue *et al*., 2014). Cadmium is easily taken up by plants and accumulates in edible parts, thus posing risks to humans health through food chains (Uraguchi and Fujiwara, 2013). Various strategies have been developed to remove Cd from polluted soils, among which phytoremediation is regarded as an environmentally friendly, cost‐effective and *in situ* remediation technology (Krämer, 2005). Although hyperaccumulators such as *Thlaspi caerulescens* are effective in extracting metals from soil (Deng *et al*., 2007; McGrath *et al*., 2006; Singer *et al*., 2007; Song *et al*., 2012), most hyperaccumulators have small biomass, slow growth rate and low economic benefit, which greatly restrict the development of phytoremediation technology (Douchiche *et al*., 2012; He *et al*., 2013).

Nowadays, it is suggested that some fast growing, metal tolerant and high biomass plant species, especially energy plants, may be more effective in phytoextraction of heavy metals from contaminated soils (Juwarkar *et al*., 2008). Sweet sorghum (*Sorghum bicolor* (L.) Moench), a C_4_ plant with high photosynthetic efficiency (Gnansounou *et al*., 2005; Yu *et al*., 2012), is resistant to stresses such as salt, drought, heat and toxic pollution. Sweet sorghum is widely cultivated in tropical, subtropical and temperate regions (Marchiol *et al*., 2007; Zhuang *et al*., 2009). In addition, it has high biomass yield and high sugar content in its stalk, making it an ideal feedstock for ethanol production (Bennett and Anex, 2009; Calvino and Messing, 2012; Gnansounou *et al*., 2005). Phytoremediation with sweet sorghum can not only combine soil remediation with bioenergy production, but also transfer heavy metals from the food chain into the energy chain, thus avoiding the harm to human beings (Jia *et al*., 2016; Li, 2013; Woods, 2001). Previous studies have shown that sorghum was more tolerant to Cd and copper (Cu) treatment than wheat and maize (Metwali *et al*., 2013). What's more, sorghum can accumulate large quantities of Cd, Cu, lead (Pb) and zinc (Zn) in shoots as its biomass was higher than that of sunflower or corn (Zhuang *et al*., 2009). However, as nonhyperaccumulator, most of the absorbed Cd was retained in the roots of sweet sorghum plants, while only a small amount can be transported to the shoots (Angelova *et al*., 2011; Jia *et al*., 2016; Soudek *et al*., 2014). To improve the capacity of phytoremediation, it is of great importance to promote the capacity of Cd accumulation in its shoots.

Several physiological processes determine the Cd accumulation in the shoots of plants, including root uptake, sequestration into vacuoles and translocation in the xylem as well as phloem. Cadmium in soil enters plants via apoplasmic and symplasmic pathways (Lu *et al*., 2009). It is generally accepted that active acquisition of Cd occurs mainly via uptake systems for essential elements, such as Fe^2+^, Ca^2+^, Zn^2+^ and Mn^2+^ (Lombi *et al*., 2002; Pence *et al*., 2000; Perfus‐Barbeoch *et al*., 2002). However, a specific mechanism of Cd uptake mediated by high‐affinity Cd transporters was also suggested in *T. caerulescens* (Zhao *et al*., 2002). After absorption by the roots, Cd is transported to the stele by passing through endodermis (Lux *et al*., 2011) and then translocated to shoot via xylem, the latter of which is driven by transpiration from leaves (Lu *et al*., 2009; Lux *et al*., 2011). Further accumulation of Cd into seeds or grains is mediated by phloem transport as illustrated by several studies (Kato *et al*., 2010; Uraguchi *et al*., 2011). Previous studies on the response of sweet sorghum to Cd stress were mostly descriptive and limited to the physiological level. Currently, the uptake, translocation and accumulation processes of Cd in this valuable energy plant are still mostly unknown. In this study, using two contrasting sweet sorghum genotypes, the mechanisms responsible for their differential Cd accumulation were investigated through a combination of physiological, microstructural and comparative transcriptome analyses.

## Results

### Sweet sorghum genotypes H18 and L69 exhibited a great difference in the capacity of Cd uptake and translocation

**Figure 1:**
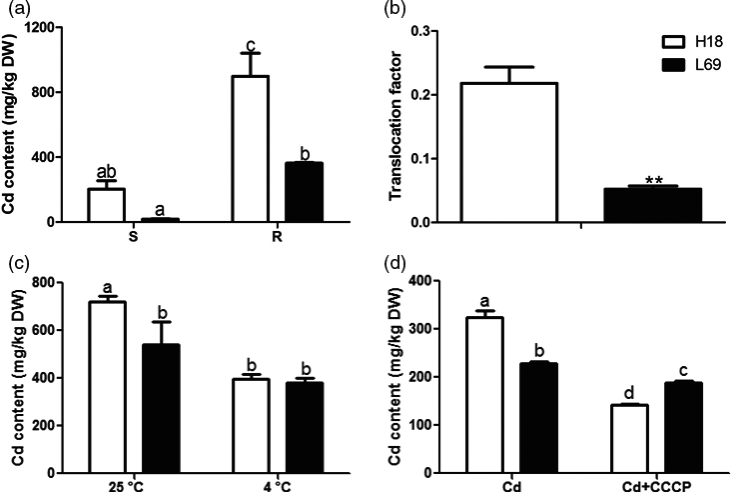
Cd uptake and accumulation was much higher in H18 than in L69. (a, b) H18 and L69 seedlings were treated with 0 and 10 μm CdCl_2_ for 2 weeks, and Cd content in shoot (S) and root (R) was detected (a), and translocation factor was calculated by (Cd content in shoot)/(Cd content in root) (b). (c, d) H18 and L69 seedlings were transferred to uptake solution with or without 10 μm CdCl_2_ at 25 and 4 °C (c) or 100 μm CCCP (d). After 24 h, Cd contents in roots were detected. Values are means ± SE (*n* = 3 and five plants for each replicate). Different letters above the bars indicate significant differences at *P *<* *0.05. For (b), double asterisks above the column indicate significant difference (Student's *t*‐tests, *P *<* *0.01).

In previous study, we identified two sweet sorghum genotypes with contrasting Cd translocation factor (TF) from ninety‐six germplasm obtained from USDA (United States Department of Agriculture), that is UMM EL TEIMAN (Accession NO. PI 152873, designated as H18), with highest TF and MN 4578 (Accession NO. PI 273969, designated as L69) with lowest TF (in submission). To compare the effects of cadmium on H18 and L69 plants, the growth parameters were first detected. Under both control and Cd conditions, the biomass of L69 was slightly higher than that of H18 (Figure S1a). Cd treatment suppressed the growth of H18 and L69 seedlings. Although the plant height reduction was comparable between H18 and L69 (Figure S1b), the dry weight reduced about 40% in L69, which was much more than that in H18 (about 15%) (Figure S1c). In addition, the photosynthetic rate and chlorophyll contents decreased after Cd treatment, both with lower values in L69 than in H18 (Figure S1d,e). Furthermore, most of the absorbed Cd was retained in the roots of H18 and L69 seedlings, yet the Cd content of root and shoot was both higher in H18 (Figure 1a). Moreover, the Cd translocation factor of H18 was about fourfold as high as L69 (Figure 1b). These results indicate that H18 has much higher Cd accumulation and translocation capacity than L69. Metals can be absorbed by plant roots both passively and actively, and thus, the effect of low temperature (4 °C) and metabolic inhibitor CCCP on Cd accumulation was investigated as symplasmic pathway was predicted to be minimal under these conditions (Zhao *et al*., 2002). Low temperature or addition of CCCP both significantly inhibited Cd uptake in H18 and L69 roots, yet the inhibitory effect was much more pronounced in H18 (Figure 1c,d). The Cd accumulation was comparable in H18 and L69 roots under 4 °C (Figure 1c) while it was significantly lower in H18 than L69 after CCCP treatment (Figure 1d). As net symplasmic uptake can be estimated by subtracting the apparent uptake at 4 °C or under CCCP treatment from that of 25 °C, these results indicated higher symplasmic Cd uptake by root in H18 than in L69.

**Figure 2:**
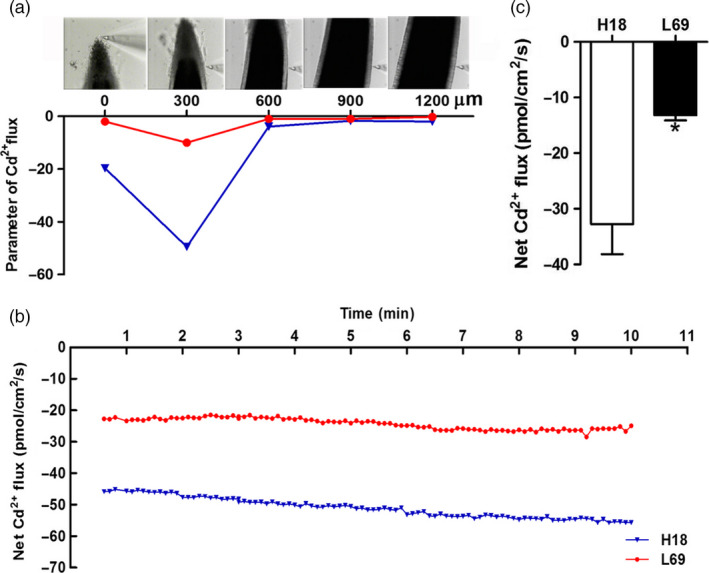
The net Cd^2+^ influx was higher in H18 roots than L69. (a) Parameter of Cd^2+^ fluxes in root cells of H18 and L69 at different distances from root tip. (b) Net Cd^2+^ fluxes in one representing root of H18 and L69, which was detected at 300 μm from the root tip for 10 min. (c) The mean Cd^2+^ fluxes in root cells within 10‐min measuring periods. Values are means ± SE (*n* = 3). Asterisk indicates significantly different from those of H18 (Student's *t*‐tests, *P *<* *0.05).

Scanning ion‐selective electrode technique (SIET) was also used to investigate Cd^2+^ influx. We scanned 0–1200 μm from root tips of H18 and L69 and detected the highest Cd influx rate at 300 μm (Figure 2a). Thus, this site was selected for Cd^2+^ influx detection in the subsequent analysis. Marked differences were found between the two genotypes while the Cd^2+^ influx value in H18 roots was 2.5‐fold of that in L69, indicating higher Cd uptake capacity in H18 roots compared to L69 (Figure 2b,c).

### Endodermal apoplasmic barriers were much stronger in L69 than those in H18

**Figure 3:**
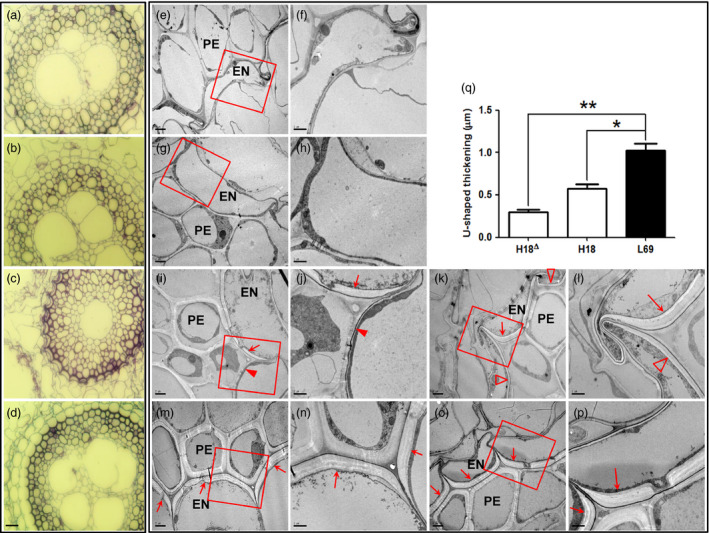
Light and transmission electron microscopy of root ultrastructure. (a–d) Light micrographs of transverse sections of H18 (a, c) and L69 (b, d) roots under CK (a, b) and 10 μm CdCl_2_ (c, d) conditions (scale bar = 20 μm). (e–p) Transmission electron microscopy of H18 (e‐f, i‐l) and L69 (g‐h, m‐p) roots under CK (e–h) and 10 μm CdCl_2_ (i–p) treatment. f, h, j, l, n and p (scale bars = 1 μm) were enlargements of boxes in e, g, i, k, m and o (scale bars = 2 μm), respectively. U‐shaped thickenings were showed by arrows, while endodermal cells without U‐shaped thickening or with thinner cell walls in H18 were showed by filled and empty triangles, respectively. PE, pericycle; EN, endodermis. (q) The quantitative results of U‐shaped thickenings in H18‐Cd (H18^∆^ and H18, corresponding to empty triangles and arrows in i‐l) and L69‐Cd samples. One and double asterisks above the bars indicate significant differences from L69 at *P *<* *0.05 and *P *<* *0.01, respectively (*n* = 3 root sections, and value of each section was averaged from more than 10 endodermal cells except H18^∆^, which was averaged from 3 cells).

After absorption by the roots, Cd is transported to the stele by passing through endodermis (Lux *et al*., 2011). We thus observed the root anatomy structure through semi‐thin and ultrathin section microscopy. Cd treatment caused considerable anatomical alterations in H18 roots (Figure 3a,c), whereas the anatomy structure of L69 changed a little (Figure 3b,d). Under Cd condition, the endodermis became falcate in H18 roots, and the xylem was more concentrated surrounding only one pole compared to three poles in control (Figure 3a,c). However, the endodermis kept normal plump shape in L69 roots and xylem differentiation was slightly affected (Figure 3d). Through transmission electron microscopy, we observed intensive U‐shaped thickenings of endodermal cells in Cd‐treated roots of H18 and L69 (Figure 3i–p). However, U‐shaped thickenings were found in all endodermal cells of L69 roots (Figure 3m–p). In contrast, in H18 roots, some endodermal cells without U‐shaped thickenings (Figure 3i,j) or with thinner cell walls (Figure 3k,l) were found. When quantified, the endodermal U‐shaped thickenings in L69 roots were significantly thicker (1.03 ± 0.14) than those in H18 roots (0.57 ± 0.09 and 0.29 ± 0.06) (Figure 3q). These results indicated that endodermal apoplasmic barriers were much stronger in L69 than those in H18 under Cd stress.

### Cd contents in xylem sap was much higher in H18 than that in L69

To examine the translocation ability of Cd into the shoot, xylem sap was collected from H18 and L69 plants. The collected xylem sap had a total protein concentration of 10–14 μg/mL (Table S1), being comparable with maize xylem sap (12 μg/mL) (Alvarez *et al*., 2006) but far lower than phloem sap values (200 μg/mL in rice and *Lupinus* species to 35–60 mg/mL in Curcubitaceae) (Rodriguez‐Celma *et al*., 2016). In addition, it contained very low concentrations of sucrose, glucose and fructose (Table S1), which was characteristic of xylem saps but was different from the phloem extrudes containing high concentrations of sucrose (238 mM in castor bean) (Hall and Baker, 1972; Smith and Milburn, 1980; Ye *et al*., 2010). Both of these results showed good purity of the collected xylem sap with very low possibility of contamination from phloem sap.

**Figure 4:**
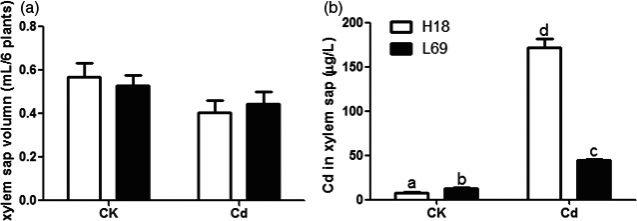
Cd concentration in the xylem sap was higher in H18 than in L69. Total volume (a) and Cd concentrations (b) of xylem sap collected from H18 and L69. Data are means ± SEs, and different letters on the bar indicate significant difference at *P *<* *0.05 (*n* = 4 and six plants for each replicate).

Then, Cd concentrations in collected xylem sap were determined. The exposure to Cd decreased xylem sap transport in both genotypes although the decline was not significant, and the volume of xylem sap in H18 and L69 was comparable both under control and Cd conditions (Figure 4a). However, the Cd concentration in xylem sap of H18 was significantly higher than that of L69, which was about twice as much as that of L69 (Figure 4b).

### Cd affected the accumulation and transport of mineral elements

Cd interferes with the accumulation of micronutrients in plants. Thus, the effects of Cd on Fe, Zn, manganese (Mn), Cu, calcium (Ca) and magnesium (Mg) accumulation in H18 and L69 plants were investigated. The changes of Fe and Cu contents had the same tendency, which increased significantly in roots while not changed in shoots, and the values in roots were higher in H18 than in L69 (Figure S2a,d). The accumulation of Zn and Mn did not change in roots while decreased in shoots in both H18 and L69 (Figure S2b,c). The contents of Ca increased in both roots and shoots of H18, but remained unchanged in L69 (Figure S2e). In addition, Mg accumulation decreased in the roots of both H18 and L69 while increased only in H18 shoots (Figure S2f).

The accumulations of Fe, Zn, Mn, Cu, Ca and Mg in xylem exudate were also detected, among which Zn and Cu contents were not affected by Cd (Figure S3b,d) while Fe and Mn contents decreased (Figure S3a) and increased (Figure S3c), respectively, only in L69. In addition, after Cd treatment, Ca content in xylem increased in H18 yet decreased in L69 (Figure S3e), while Mg was only up‐regulated in H18 (Figure S3f).

### Comparative transcriptome analysis identified key genes and processes responsible for differential Cd accumulation between H18 and L69

To further elucidate the molecular basis for the differential Cd accumulation in H18 and L69, we conducted comparative transcriptome analysis through high‐throughput digital gene expression (DGE) sequencing. A 24‐h treatment of 10 μm Cd exposure was used to investigate early response of sweet sorghum to Cd stress. Twelve DGE libraries were created from three biological replicates each for H18 and L69 roots under control and CdCl_2_ treatments and sequenced. After removing low‐quality reads and those containing adapter and poly‐N, more than 10 million clean reads remained in each sample (Table S2), among which more than 90% was mapped to the *Sorghum bicolor* genome (http://phytozome.jgi.doe.gov/pz/portal.html#!info?alias=Org_Sbicolor_er) (Table S3).

**Figure 5:**
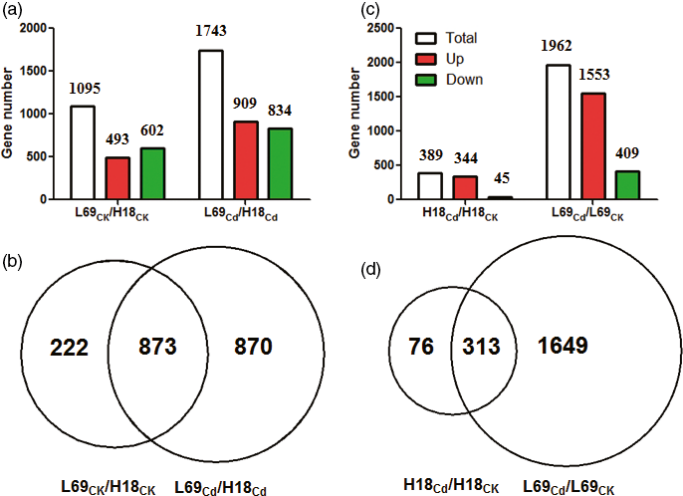
Summary of DEGs. (a) and (c), Numbers of DEGs between H18 and L69 under CK and CdCl_2_ conditions (a) and DEGs following CdCl_2_ exposure (c). (b) and (d), Venn diagrams of DEGs in (a) and (c), respectively.

Then, differentially expressed genes (DEGs) were identified through comparisons of the FPKM values for each gene between H18 and L69 (L69_CK_/H18_CK_ and L69_Cd_/H18_Cd_) or between Cd‐treated and CK samples in each genotype (H18_Cd_/H18_CK_ and L69_Cd_/L69_CK_), and thus, DEGs between two genotypes and DEGs involved in Cd response were screened, respectively. Under normal conditions, 1095 genes expressed differentially between H18 and L69, while this value reached 1743 after Cd treatment, among which 873 genes were common ones, indicating genetic differences between the two genotypes (Figure 5a,b, Table S4). For Cd‐responsive DEGs, a total of 389 genes were differentially expressed in H18 after Cd treatment, including 344 up‐regulated and 45 down‐regulated genes. However, 1962 Cd‐responsive DEGs were found in L69, including 1553 up‐regulated and 409 down‐regulated ones (Figure 5c). Among them, 313 genes were common Cd‐responsive genes (Figure 5d).

To evaluate the validity of deep‐sequencing data, five Cd‐responsive genes were selected for expression levels examination by qRT‐PCR (Table S11). The results were consistent with that of deep sequencing, with a positive correlation (*R* ^2^ = 0.6989, *P *=* *0.003) indicating the reliability of high‐throughput data (Figure S4).

#### Gene ontology (GO) analysis of DEGs

**Figure 6:**
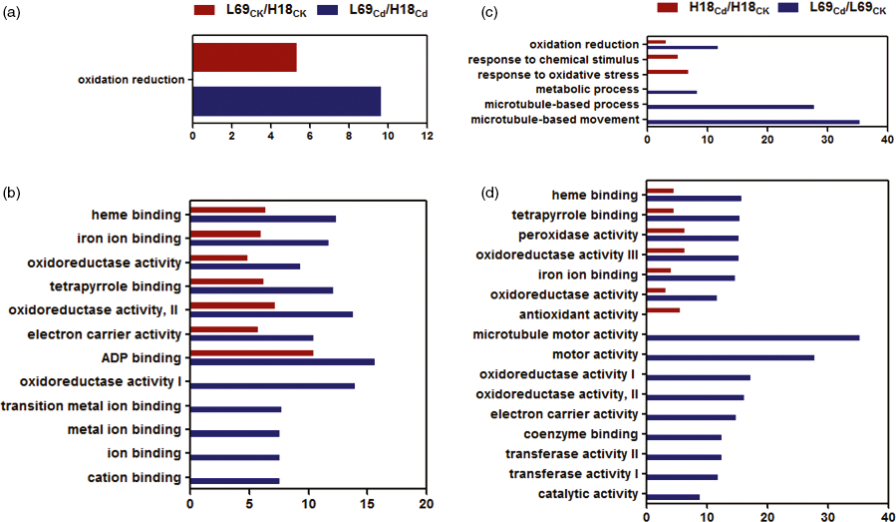
GO enrichment analysis of all DEGs. The enriched biological process GO terms (a and c) and molecular function GO terms (b and d) of DEGs between H18 and L69 under CK and CdCl_2_ conditions (a and b) and DEGs following CdCl_2_ exposure (c and d). The *x*‐axis indicates the percentage of DEGs numbers *vs*. background gene numbers in each GO term. The detailed information was shown in Table S5.

To identify the major functional categories represented by the DEGs, GO enrichment analysis was carried out using the singular enrichment analysis (SEA) in agriGO program (Du *et al*., 2010). For DEGs between H18 and L69, GOs associated with oxidation reduction in the biological process category (Figure 6a) and the ones associated with heme binding, iron ion binding, oxidoreductase activity, tetrapyrrole binding, electron carrier activity as well as ADP binding in the molecular function category (Figure 6b) were significantly enriched under both control and Cd stress conditions. For Cd‐responsive DEGs, GO items of oxidation reduction in the biological process category (Figure 6c), and heme binding, tetrapyrrole binding, peroxidase activity, oxidoreductase activity as well as iron ion binding in the molecular function category (Figure 6d) were enriched in both H18 and L69. Besides, response to chemical stimulus and response to oxidative stress in the biological process category, and antioxidant activity in the molecular function category were unique terms associated with H18 (Figure 6c,d). In L69, those of microtubule‐based movement and process as well as metabolic process (Figure 6c) in the biological process category and microtubule motor activity, motor activity, oxidoreductase activity, electron carrier activity, coenzyme binding, transferase activity and catalytic activity in the molecular function category (Figure 6d) were uniquely enriched (Table S5).

#### KEGG pathway enrichment of DEGs

**Figure 7:**
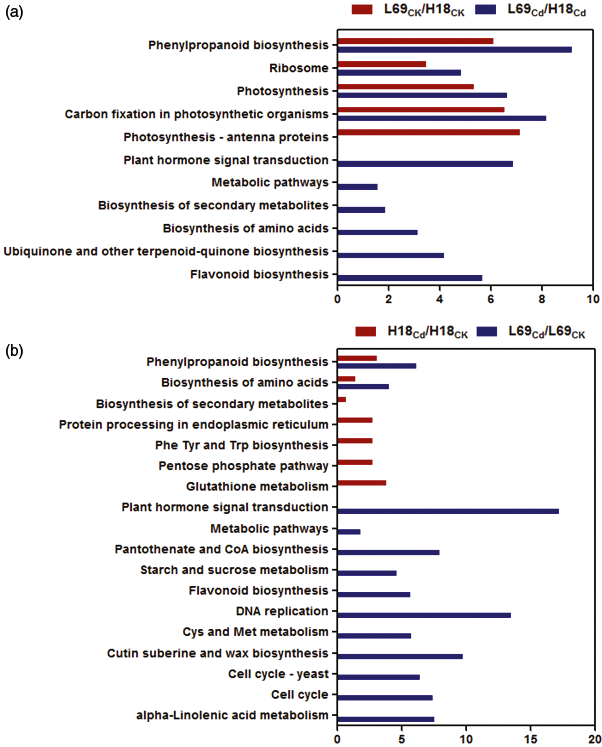
KEGG analysis of DEGs between H18 and L69 under CK and CdCl_2_ conditions (a) and DEGs following CdCl_2_ exposure (b). The *x*‐axis indicates the percentage of DEGs numbers *vs*. background gene numbers in each KEGG pathway. The detailed information is shown in Table S6.

To further systematically understand the molecular interactions among the DEGs, we performed KEGG analysis (Kanehisa and Goto, 2000). For DEGs between H18 and L69, pathways of phenylpropanoid biosynthesis, carbon fixation in photosynthetic organisms, photosynthesis and ribosome were enriched under both control and Cd conditions (Figure 7a, Table S6). Cd‐responsive DEGs in both H18 and L69 were enriched in phenylpropanoid biosynthesis and biosynthesis of amino acids pathways. Besides, the protein processing in endoplasmic reticulum, glutathione metabolism, phenylalanine, tyrosine and tryptophan biosynthesis, pentose phosphate pathway, as well as biosynthesis of secondary metabolites were specifically enriched for Cd‐responsive DEGs in H18. In contrast, plant hormone signal transduction, DNA replication, cell cycle, cysteine and methionine metabolism, metabolic pathways, pantothenate and CoA biosynthesis, alpha‐linolenic acid metabolism, cutin, suberin and wax biosynthesis, starch and sucrose metabolism as well as flavonoid biosynthesis were uniquely enriched for Cd‐responsive DEGs in L69 (Figure 7b, Table S6).

#### Genes involved in phenylpropanoid and lignin biosynthesis

**Figure 8:**
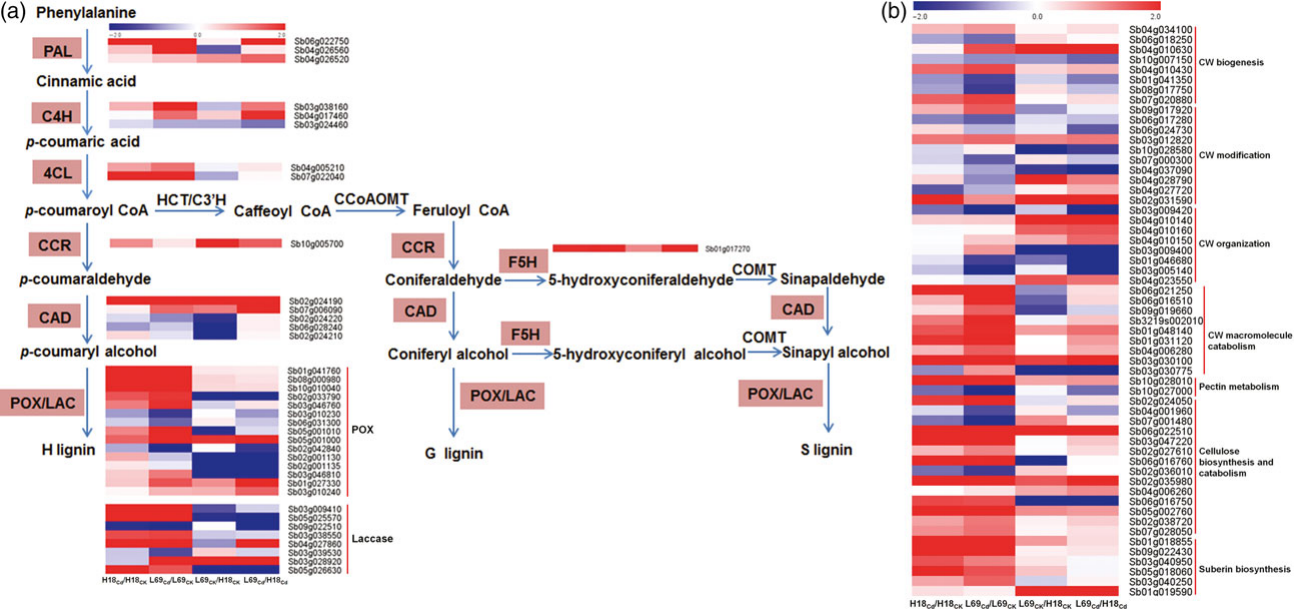
Heat map of DEGs involved in phenylpropanoid pathway and lignin biosynthesis (a) as well as cell wall modification (b). Relative expression was calculated by Log_2_Ratio. H18 _CK_ , H18_Cd_, L69 _CK_ and L69_Cd_ represent the H18 and L69 roots treated with 0 and 10 μm CdCl_2_, respectively. The detailed information is shown in Tables S7 (a) and S8 (b).

KEGG analysis showed phenylpropanoid biosynthesis was over‐presented both for Cd‐responsive DEGs and DEGs between two genotypes (Figure 7), indicating that this pathway may play important roles in Cd response of sweet sorghum and the differential Cd accumulation between H18 and L69. We thus focused on the 38 DEGs involved in this pathway, among which 13 were Cd‐responsive DEGs, 15 were DEGs between H18 and L69, while the remaining 10 were not only Cd‐responsive but also differentially expressed between the two genotypes ((Figure 8a, Table S7).

#### Other genes involved in cell wall (CW) metabolism

CW is the primary structure directly exposed to Cd, especially in roots. The transcriptome data identified other 57 DEGs involved in CW metabolism and modification (Figure 8b) besides the aforementioned 38 DEGs involved in lignin biosynthesis (Figure 8a), including seven involved in CW biogenesis, 10 involved in CW modification, eight involved in CW organization, nine involved in CW macromolecule catabolic process, two involved in pectin metabolic process, 14 involved in cellulose biosynthesis and catabolism, as well as six involved in suberin biosynthesis (Figure 8b). Among these 57 DEGs, 32 were Cd‐responsive DEGs and 18 were DEGs between H18 and L69. For the 32 Cd‐responsive DEGs, 22 were up‐regulated by Cd and 10 were down‐regulated, and most (26 of 32) were specifically responsive to Cd in L69 roots. The remaining seven genes were Cd‐responsive DEGs, which also expressed differentially between two genotypes. Interestingly, six of them were expressed higher in L69 (Table S8).

#### Genes involved in heavy metal transport

**Figure 9:**
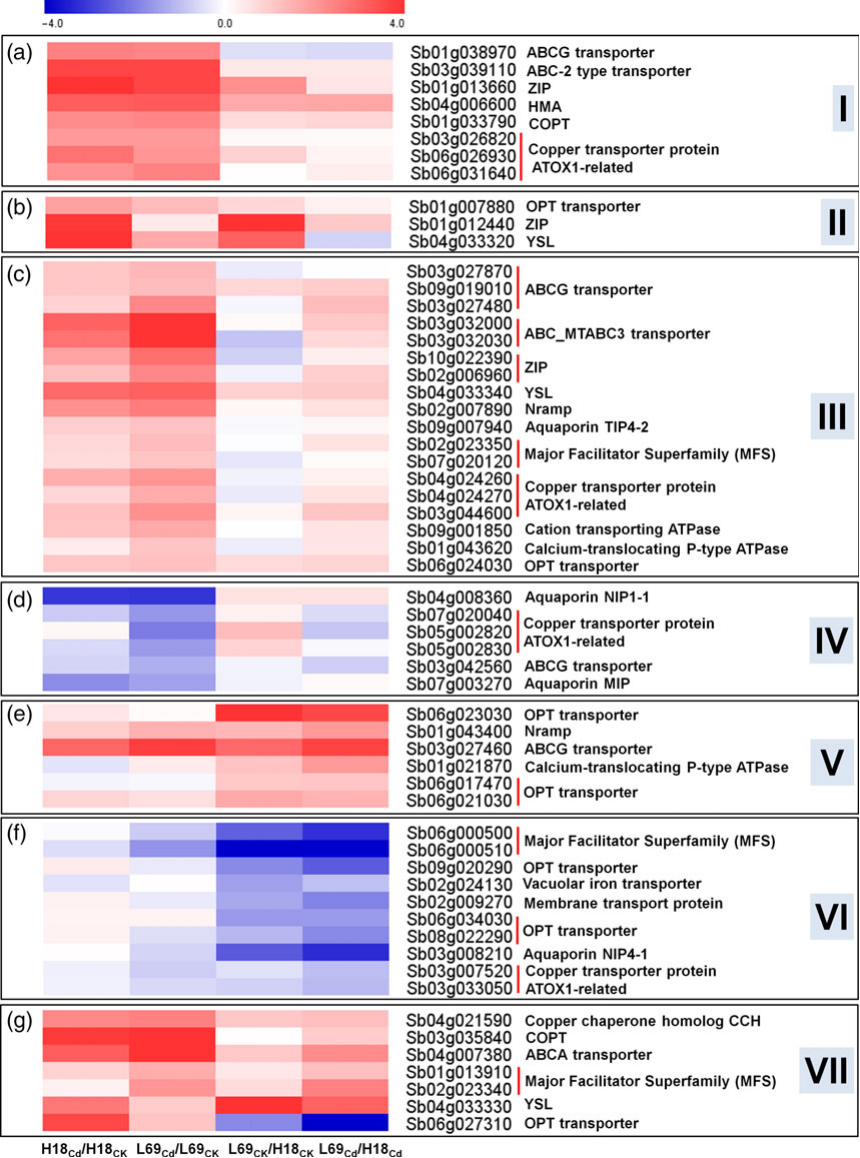
Heat map of DEGs encoding heavy metal transporters. Relative expression was calculated by Log_2_Ratio. H18 _CK_ , H18_Cd_, L69 _CK_ and L69_Cd_ represent the H18 and L69 roots treated with 0 and 10 μm CdCl_2_, respectively. The detailed information is shown in Table S9.

As transporters play important roles in Cd uptake, transport and sequestration process, 59 DEGs encoding heavy metal transporters were also examined, which can be divided into seven groups (I–VII) based on their expression patterns (Figure 9, Table S9). The first group consisted of eight genes whose expressions were significantly up‐regulated in both H18 and L69 roots under Cd treatment (Figure 9a). The three genes in the second group and the eighteen genes in the third group were specifically up‐regulated in H18 and L69 roots by Cd treatment, respectively (Figure 9b,c). Genes in the fourth group were down‐regulated by Cd treatment either in both genotypes or specifically in L69 (Figure 9d). The expression of genes in the fifth group was higher in L69 roots than those in H18 (Figure 9e). The sixth group had eleven genes expressed higher in H18 than those in L69 (Figure 9f). Groups I to IV were Cd‐responsive DEGs whereas groups V and VI were DEGs between two genotypes. The seven genes in Group VII were Cd‐responsive DEGs between H18 and L69. Three genes separately encoding copper chaperone homolog (CCH), copper transporter (COPT) and ABCA transporter were up‐regulated by Cd in both H18 and L69. Moreover, their expressions were higher in L69 than in H18. Two major facilitator superfamily genes were up‐regulated by Cd specifically and expressed higher in L69 roots. Genes encoding a yellow stripe‐like protein (YSL) and an OPT transporter were up‐regulated by Cd only in H18 roots, and YSL expressed higher in L69 while OPT expressed higher in H18, respectively (Figure 9g).

## Discussion

As an ideal feedstock for ethanol production, sweet sorghum has great potential in phytoremediation of Cd‐polluted soils (Jia *et al*., 2016; Li, 2013; Woods, 2001). However, progress in improving phytoremediation ability of this species is limited as the regulatory mechanisms of Cd uptake, accumulation and transport in sweet sorghum remain largely unexplored. Two sweet sorghum genotypes, H18 and L69, with contrasting Cd translocation factors were used in this study, with H18 collected from Sudan while L69 from Ethiopia. Although significant genetic differences were found as more than 1000 DEGs were identified between them (Figure 5), through comparing Cd‐responsive genes in H18 and L69 and identifying Cd‐responsive DEGs that expressed differentially between them, key factors for differential Cd accumulation were identified, which were consistent with the morpho‐physiological results.

### Transporters are involved in differential root uptake and xylem translocation capacity of Cd between H18 and L69

Based on the physiological data (Figures 1, 2 and 4), we reasoned that differential root uptake and xylem loading capacity of Cd may be main factors determining the contrasting Cd accumulation in H18 and L69. Uptake of Cd from the external solution to root cells and following translocation via xylem is thought to be mediated through transporters, but transporters for Cd have not been identified in sweet sorghum. Several studies have reported that transporters for essential elements such as Fe^2+^, Zn^2+^ and Ca^2+^ may be involved in Cd uptake and transport (Lux *et al*., 2011). In total, we identified 59 transporter‐encoding DEGs, among which 35 were Cd‐responsive DEGs and seven were Cd‐responsive DEGs which were also expressed differentially between H18 and L69 (Figure 9, Table S9).

**Figure 10:**
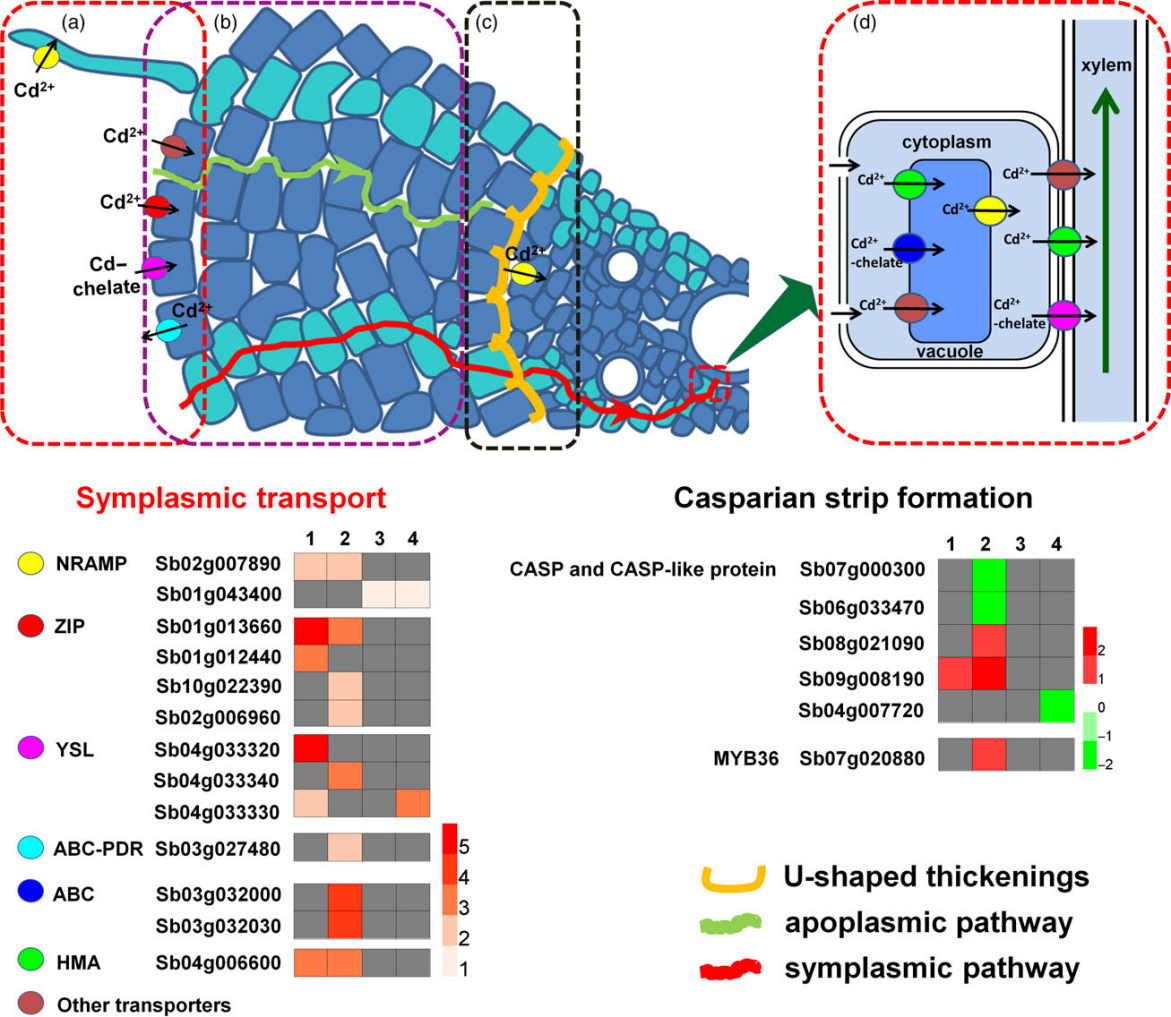
A schematic representation of main processes involved in differential Cd uptake and translocation in H18 and L69 plants. These processes include uptake of Cd from the external solution to root cells (a), cell wall binding of Cd (b), apoplastic barriers in the endodermis (c) and Cd translocation via xylem (d). Transporter genes involved in symplasmic pathway and genes involved in Casparian strip formation were shown. Numbers 1, 2, 3 and 4 represent relative expression calculated by Log_2_(H18_Cd_/H18 _CK_ ), Log_2_(L69_Cd_/L69 _CK_ ), Log_2_(L69 _CK_ /H18 _CK_ ) and Log_2_(L69_Cd_/H18_Cd_), respectively. Grey box represents the expression of the gene was not changed significantly.

Cd can enter root cells in the form of Cd^2+^ through ZIP (Zinc‐regulated transporter/iron‐regulated transporter‐like protein) transporters, which are capable of transporting a variety of divalent cations, including Cd^2+^, Fe^2+^, Zn^2+^ and Mn^2+^ (Guerinot, 2000; Plaza *et al*., 2007). In this study, four ZIP encoding genes were found to be DEGs, among which *Sb01g013660* was induced by Cd in both genotypes, *Sb01g012440* was induced by Cd only in H18, while *Sb02g006960* and *Sb10g022390* were specifically induced by Cd in L69 (Figure 10). This result indicates that uptake of Cd in H18 and L69 roots may involve different ZIP transporters.

Nramp (natural resistance‐associated macrophage protein) family members function as general metal ion transporters which can transport Fe^2+^, Zn^2+^, Mn^2+^, Cu^2+^, Ni^2+^, Co^2+^ and Cd^2+^ (Nevo and Nelson, 2006). For example, OsNramp5 functions as a major transporter responsible for root Mn and Cd uptake in rice (Sasaki *et al*., 2012). A very recent study reported that HvNramp5 is a transporter for Mn and Cd uptake in barley, which is localized in plasma membrane and constitutively expressed in the root tip epidermal cells (Wu *et al*., 2016). In this study, we found about fourfold and 1.5‐fold up‐regulated expression of *SbNramp5* (*Sb02g007870*) by Cd treatment in H18 and L69, respectively; however, it was not DEGs as judged by our criteria. This result is similar to HvNramp5, whose expression is not affected by Cd despite it shows transport ability for Cd^2+^ (Wu *et al*., 2016). In addition, report on OsNramp1 showed that it might assist xylem loading of Cd for root to shoot mobilization (Tiwari *et al*., 2014). A *SbNramp* gene (*Sb02g007890*) homologous to *OsNramp1* was up‐regulated under Cd stress in both H18 and L69 roots, indicating that it might be involved in Cd translocation in sweet sorghum. However, no difference in expression was found for *Sb02g007890* between the two genotypes. Besides, higher expression of another *SbNramp* gene (*Sb01g043400*) homologous to *AtNramp2/3/4/6* was detected in L69 both constitutively and under Cd stress. AtNramp3 and AtNramp4 were localized in vacuolar membrane and function in export of metals (Fe, Zn, and Mn) from the vacuolar compartment to the cytosol (Lanquar *et al*., 2004, 2005, 2010; Thomine *et al*., 2003). AtNramp6 functions as an intracellular metal transporter, whose presence, when modified, is likely to affect the distribution of Cd within the cell. *AtNramp6* over‐expressed Arabidopsis plants were hypersensitive to Cd, although Cd content remained unchanged (Cailliatte *et al*., 2009). We suggest that *Sb01g043400* may play important roles in Cd tolerance through export of Cd from vacuolar to cytosol in sweet sorghum. However, the exact role needs to be further investigated.

In addition to Cd^2+^, Cd can enter root cells as Cd‐chelates through YSL (yellow stripe 1‐like) proteins (Curie *et al*., 2009), which are also involved in the loading of Cd from symplasm into xylem (Verbruggen *et al*., 2009). In this study, three YSL‐encoding genes (*Sb04g033320*,* Sb04g033330* and *Sb04g033340*) were identified to be DEGs (Figure 10). Interestingly, the former two were induced by Cd only in roots of H18, the high‐Cd accumulation genotype, and the expression of *Sb04g033320* was up‐regulated for even more than 40‐fold. We inferred that these genes may play essential roles in Cd uptake and/or xylem loading in genotype H18. Further study of the exact molecular function may help reveal the mechanism of differential Cd accumulation in the two sweet sorghum genotypes.

P_1B_‐type ATPase (heavy metal transporting ATPase, HMA) proteins also play important roles in xylem loading of Cd. Arabidopsis has eight members of HMA family proteins, among which AtHMA3 is involved in sequestration of Zn and Cd into vacuoles (Williams and Mills, 2005), while AtHMA2 and AtHMA4 are implicated in xylem loading of Cd and Zn (Hanikenne *et al*., 2008; Mills *et al*., 2003; Papoyan and Kochian, 2004; Verret *et al*., 2004). Through BLASTP analysis, we identified 10 candidate genes encoding HMA in *S. bicolor* genome (Table S10). However, only one of them, *Sb04g006600*, was found to be induced by Cd treatment in both genotypes and its expression was comparable between H18 and L69. This result indicated that although *Sb04g006600* may play roles in Cd response in sweet sorghum, its involvement in differential Cd accumulation in H18 and L69 may not be based on the regulation of expression but on the different functions, just as the recent report on OsHMA3. Yan *et al*. found a loss‐of‐function allele of *OsHMA3* with a predicted amino acid mutation from Ser to Arg at the 380th position, which was associated with high‐Cd accumulation in shoots and grain of a *Japonica* rice cultivar (Yan *et al*., 2016).

### Enhanced cell wall (CW) binding and endodermal apoplasmic barriers in L69 may restrict Cd translocation to shoot

CW is the first barrier to toxic metals in the environment, whose synthesis and composition are affected by heavy metals (Douchiche *et al*., 2010; Fernández *et al*., 2014; Sun *et al*., 2013; Wan and Zhang, 2012). Here, we found the apoplasmic Cd uptake was significantly lower in H18 than in L69 (Figure 1d). Although Cd concentration in CW was a little lower in L69, the CW yield in L69 was much higher under Cd treatment (Figure S5), resulting in higher total Cd content in L69 root CWs. As CW is the major pool of heavy metal accumulation in roots (Fernández *et al*., 2014), a higher binding capacity of CW to Cd in L69 will result in a lower distribution of Cd to the shoot. After uptake by roots, the transport of Cd to shoot is controlled by apoplasmic barriers in endodermis (Schreiber, 2010), whose differentiation generally includes three stages: the formation of Casparian strips, suberin lamellae and U‐shaped tertiary walls (Meyer *et al*., 2009; Redjala *et al*., 2011). Exposure to Cd can induce acclamatory responses of endodermis, including extensive thickening of the inner tangential walls of endodermal cells (Degenhardt and Gimmler, 2000), formation of Casparian strips and suberin lamellae closer to the root apex (Martinka and Lux, 2004; Schreiber *et al*., 1999; Vaculík *et al*., 2009; Zelko and Lux, 2004), increase of suberin and lignin contents as well as the alteration of their chemical composition (Schreiber *et al*., 1999). All these changes can reduce apoplasmic movement of Cd to xylem and its translocation to shoot. In this study, U‐shaped tertiary walls were found in endodermis of both H18 and L69 roots under Cd stress (Figure 3i–p), indicating that Cd treatment could induce and enhance the suberization and lignification of the endodermis. It is worth noting that the endodermal apoplasmic barriers was much stronger in L69 roots, as the tertiary walls of endodermis were significantly thicker in L69 (Figure 3q) and there were no visible passage cells in L69 (Figure 3m–p) in contrast to many passage cells in H18 roots (Figure 3i,j). Passage cells are endodermal cells with unsuberized tangential walls, usually found opposite the xylem poles. Although the exact functions are uncertain, available evidence suggests that passage cells are important for transfer of Ca and Mg into stele and thus into the transpiration stream (Peterson and Enstone, 1996). We proposed that the stronger endodermal apoplasmic barriers in L69 can prevent a higher proportion of Cd through apoplasmic transport into xylem. In contrast, passage cells in H18 endodermis may render the transport of Cd into stele more efficiently.

In accordance with the morpho‐physiological results, many DEGs identified by transcriptome data were found to be linked with CW modification, including genes involved in CW biogenesis and modification as well as CW macromolecule (pectin, cellulose, lignin and suberin) catabolic process. In addition, KEGG analysis showed over‐representation of phenylpropanoid biosynthesis pathway, the product of which serves as metabolite source for lignin biosynthesis (Vogt, 2010). Interestingly, most of these DEGs were specifically Cd‐responsive DEGs in L69 or expressed higher in L69, including 21 of 38 DEGs involved in lignin biosynthesis, 42 of the other 57 DEGs involved in cell wall modification, four of six DEGs involved in suberin biosynthesis, as well as four of six DEGs involved in formation of Casparian strip (Figures 8 and 10, Tables S7 and S8). We inferred that the composition or the cross‐linking of varied CW components might be different between H18 and L69 roots, resulting in different binding capacity of Cd to CW. On the other hand, discriminating ultrastructure of apoplasmic barriers in endodermis, imposed by differences in biological processes such as lignin and suberin biosynthesis and formation of Casparian strip, may also contribute to differential Cd uptake and translocation.

Based on the above results and previous studies, a schematic representation of main processes involved in differential Cd uptake and translocation in H18 and L69 plants was proposed (Figure 10). Our results suggest that higher Cd accumulation in sweet sorghum genotype H18 than in L69 depends on a multilevel coordination of efficient Cd uptake and transport, which is determined by efficient root uptake and xylem loading, less root cell wall binding, and weaker endodermis apoplasmic barriers. This study can not only lay the foundation for further exploring the molecular mechanisms of Cd accumulation in plants, but also provide new strategies for improving phytoremediation ability of energy plants through genetic engineering.

## Experimental procedures

### Plant materials and growth conditions

Sweet sorghum genotypes H18 and L69 were obtained from Plant Genetic Resources Conservation Unit, USDA, Griffin (http://www.ars-grin.gov/npgs/index.html). Sweet sorghum plants were grown hydroponically according to the conditions used by Jia *et al*. (2016).

### Low temperature and metabolic inhibitor treatment

Two‐week‐old hydroponically grown H18 and L69 seedlings were transferred to uptake solution containing 0.5 mM CaCl_2_ and 2 mM MES (pH 5.8) with or without 10 μm CdCl_2_ for different treatments including: control (25 °C), low temperature (4 °C) and Cd + 100 μm carbonyl cyanide *m*‐chlorophenylhydrazone (CCCP). Before the uptake experiment at 4 °C started, plants were pretreated with an ice‐cold uptake solution for 30 min and uptake containers were placed in an ice bath. After 24 h, plants were rinsed with uptake solution, which were used to determine Cd concentrations.

### Determination of Cd and other micronutrients contents

The roots of H18 and L69 seedlings were washed with distilled water to remove surface ions, and then, roots and shoots were harvested separately, which were dried at 70 °C until constant weight, and were grounded to fine power and digested in 2 mL nitric acid at 180 °C for 4 h. The concentrations of Cd and mineral elements were determined using ICP‐AES (Thermo Fisher Scientific, 81 Wyman Street, Waltham, MA, USA).

### Root anatomical structure and ultrastructure observation

Ultrathin sections of roots were obtained as described by Jia *et al*. (2016) and observed under a JEM‐1230 electron microscope (JEOL, Tokyo, Japan). The semi‐thin sections (1 mm) were cut using an ultramicrotome (Leica Microsystems, Wetzlar, Germany) and were stained with 1% (w/v) toluidine blue in 1% (w/v) borax. Images were taken using a Zeiss Axioplan II compound microscope with a Maximal Ratio Combiner digital camera and AxioVision software (Carl Zeiss, Jena, Germany).

### Measurement of Cd^2+^ flux

The net Cd^2+^ flux was measured using SIET (BIO‐001A; Younger USA Sci. & Tech. Corp., Beijing, China) as described by Xu *et al*. (2012).

### Collection of xylem sap and detection of Cd and mineral elements contents

Xylem sap was collected as described previously (Deng *et al*., 2013; Lu *et al*., 2013; Ueno *et al*., 2008; Wu *et al*., 2015). H18 and L69 seedlings were treated by 10 μm CdCl_2_ for 24 h, which were de‐topped at 2 cm above the shoot‐root junction. The surface of the excised leaf sheath was gently wiped, and after discarding the initial 1–2 μL of exudates, a tube filled with cotton was placed on the cut end and wrapped with Parafilm. The xylem sap was collected for 8 h, which was then weighed and stored at −20 °C until further analysis. Twenty‐four plants were used for xylem sap collection, and six individual samples each were pooled at random into four tubes. Forty microliter xylem samples were diluted to 10 mL, and the contents of Cd and mineral element were analysed by ICP‐MS or ICP‐AES.

### Digital gene expression (DGE) tag sequencing

#### cDNA library construction and sequencing

Two‐week‐old H18 and L69 seedlings were treated with 0 (CK) and 10 μm CdCl_2_ (Cd) for 24 h. Then, roots of eight seedlings were collected for RNA extraction. Each treatment had three biological replicates, resulting in a total of 12 samples. Total RNA was extracted with RNAiso™ plus (TaKaRa Bio Inc., Otsu, Shiga, Japan) and treated with RNase‐free DNase I (Fermentas, Thermo Scientific, Waltham, MA) to remove the residual DNA. The quantity of RNA was checked using the NanoDrop ND‐1000 (Thermo Scientific, Wilmington, DE) and 1.2% agarose gels. The integrity of the total RNA was assessed using Agilent 2200 Tape Station (Agilent Technologies, Santa Clara, CA), and each sample had an RNA integrity number (RIN) >8.0.

cDNA libraries were constructed using the Illumina Truseq RNA sample preparation Kit (Illumina Inc., San Diego, CA) following the manufacturer's recommendations, which were then sequenced by the Illumina HiSeq™ 2500 platform (RiboBio Co. Ltd, Guangzhou, China).

#### Sequencing data analysis—Quality control

The raw data were first filtered through in‐house Perl scripts to obtain high‐quality clean reads. Simultaneously, the Q20, Q30 and GC content levels of the clean data were evaluated. The data sets generated and/or analysed during the current study are available in the NCBI SRA repository under accession number SRP080196 (http://www.ncbi.nlm.nih.gov/sra/SRP080196).

#### Reads mapping and gene annotation

The reference genome and gene model annotation files were directly downloaded from NCBI. The FM index of the reference genome was built using Bowite (Broad Institute, Cambridge, MA). The clean reads were then aligned to the reference genome using TopHat (Broad Institute). During the alignment of reads, seven mismatches and a gap length of 7 bp were allowed. The functional annotations of genetic variants were generated using ANNOVAR.

#### Quantification of gene expression level

Gene expression levels were measured via RNA‐seq analysis as a unit of expected number of fragments per kilobase of transcript sequence per million base pairs sequenced (FPKM) (Trapnell *et al*., 2010). DESeq software was used to identify DEGs (Anders and Huber, 2010). The DESeq analysis was performed by fitting normalized count data to a generalized linear model (GLM) estimating a negative binomial distribution of the calculated mean values of the three biologically independent samples. The sequences with fold change > 2 and *P*_adj_ < 0.05 (*P* values adjusted for multiple testing with the Benjamini‐Hochberg procedure) were deemed to be DEGs.

#### GO and KEGG pathway analyses

GO enrichment analysis was performed by the agriGo program (http://bioinfo.cau.edu.cn/agriGO/). The GO terms in SDEGs with corrected *P *<* *0.05 were defined as significantly enriched. KEGG pathway analysis was carried out using the KOBAS software (KOBAS, Surrey, UK).

### Statistical analysis

One‐way ANOVA were performed using the SPSS 17.0 program. Differences were tested by Duncan's test unless otherwise specified.

## Supporting information


**Figure S1** Effects of cadmium on the growth of H18 and L69 seedlings.

**Figure S2** Effects of Cd treatment on micronutrients accumulation.

**Figure S3** Effects of Cd treatment on micronutrients contents in xylem sap.

**Figure S4** qRT‐PCR validation of expression profiles of DEGs.

**Figure S5** The effect of Cd treatment on cell wall yield (a) and Cd concentration in CW (b) in H18 and L69 roots.

**Table S1** The total protein concentrations and sugar composition in xylem exudates of H18 and L69 plants.

**Table S2** Sequencing output statistics.

**Table S3** Mapping of RNA‐seq reads to the reference *S. bicolor* genome sequence.

**Table S4** List of differentially expressed genes (DEGs).

**Table S5** Enriched GO categories of the DEGs.

**Table S6** KEGG enrichment results of the DEGs.

**Table S7** DEGs involved in phenylpropanoid and lignin biosynthesis.

**Table S8** DEGs involved in cell wall metabolism.

**Table S9** DEGs encoding possible heavy metal transporters.

**Table S10** The *HMA* genes in *S. bicolor* genome and their expression profiles in H18 and L69 roots under control and 10 μm CdCl_2_ conditions.

**Table S11** The primers used in qRT‐PCR analysis.

**Appendix S1** Supplemental methods.

## References

[pbi12795-bib-0001] Alvarez, S. , Goodger, J.Q. , Marsh, E.L. , Chen, S. , Asirvatham, V.S. and Schachtman, D.P. (2006) Characterization of the maize xylem sap proteome. J. Proteome Res. 5, 963–972. 16602704 10.1021/pr050471q

[pbi12795-bib-0002] Anders, S. and Huber, W. (2010) Differential expression analysis for sequence count data. Genome Biol. 11, R106. 20979621 10.1186/gb-2010-11-10-r106 PMC3218662

[pbi12795-bib-0003] Angelova, V.R. , Ivanova, R.V. , Delibaltova, V.A. and Ivanov, K.I. (2011) Use of sorghum crops for *in situ* phytoremediation of polluted soils. J. Agric. Sci. Technol. A. 1, 693–702.

[pbi12795-bib-0004] Bennett, A.S. and Anex, R.P. (2009) Production, transportation and milling costs of sweet sorghum as a feedstock for centralized bioethanol production in the upper Midwest. Bioresour. Technol. 100, 1595–1607. 18951018 10.1016/j.biortech.2008.09.023

[pbi12795-bib-0005] Cailliatte, R. , Lapeyre, B. , Briat, J.F. , Mari, S. and Curie, C. (2009) The NRAMP6 metal transporter contributes to cadmium toxicity. Biochem. J. 422, 217–228. 19545236 10.1042/BJ20090655

[pbi12795-bib-0006] Calvino, M. and Messing, J. (2012) Sweet sorghum as a model system for bioenergy crops. Curr. Opin. Biotechnol. 23, 323–329. 22204822 10.1016/j.copbio.2011.12.002

[pbi12795-bib-0007] Curie, C. , Cassin, G. , Couch, D. , Divol, F. , Higuchi, K. , Le Jean, M. , Misson, J. *et al*. (2009) Metal movement within the plant: contribution of nicotianamine and yellow stripe 1‐like transporters. Ann. Bot. 103, 1–11. 18977764 10.1093/aob/mcn207 PMC2707284

[pbi12795-bib-0008] Degenhardt, B. and Gimmler, H. (2000) Cell wall adaptations to multiple environmental stresses in maize roots. J. Exp. Bot. 51, 595–603. 10938816 10.1093/jexbot/51.344.595

[pbi12795-bib-0009] Deng, D.M. , Shu, W.S. , Zhang, J. , Zou, H.L. , Lin, Z. , Ye, Z.H. and Wong, M.H. (2007) Zinc and cadmium accumulation and tolerance in populations of *Sedum alfredii* . Environ. Pollut. 147, 381–386. 16828210 10.1016/j.envpol.2006.05.024

[pbi12795-bib-0010] Deng, F. , Yamaji, N. , Xia, J. and Ma, J.F. (2013) A member of the heavy metal P‐type ATPase OsHMA5 is involved in xylem loading of copper in rice. Plant Physiol. 163, 1353–1362. 24064929 10.1104/pp.113.226225 PMC3813655

[pbi12795-bib-0011] Douchiche, O. , Soret‐Morvan, O. , Chaïbi, W. , Morvan, C. and Paynel, F. (2010) Characteristics of cadmium tolerance in ‘Hermes’ flax seedlings: contribution of cell walls. Chemosphere, 81, 1430–1436. 20884040 10.1016/j.chemosphere.2010.09.011

[pbi12795-bib-0012] Douchiche, O. , Chaïbi, W. and Morvan, C. (2012) Cadmium tolerance and accumulation characteristics of mature flax, cv. Hermes: contribution of the basal stem compared to the root. J. Hazard. Mater. 235–236, 101–107. 10.1016/j.jhazmat.2012.07.027 22858130

[pbi12795-bib-0013] Du, Z. , Zhou, X. , Ling, Y. , Zhang, Z.H. and Su, Z. (2010) AgriGO: a GO analysis toolkit for the agricultural community. Nucleic Acids Res. 38, W64–W70. 20435677 10.1093/nar/gkq310 PMC2896167

[pbi12795-bib-0014] Fernández, R. , Fernández‐Fuego, D. , Bertrand, A. and González, A. (2014) Strategies for Cd accumulation in *Dittrichia viscosa* (L.) Greuter: role of the cell wall, non‐protein thiols and organic acids. Plant Physiol. Biochem. 78, 63–70. 24636908 10.1016/j.plaphy.2014.02.021

[pbi12795-bib-0015] Gnansounou, E. , Dauriat, A. and Wyman, C.E. (2005) Refining sweet sorghum to ethanol and sugar: economic trade‐offs in the context of North China. Bioresour. Technol. 96, 985–1002. 15668196 10.1016/j.biortech.2004.09.015

[pbi12795-bib-0016] Guerinot, M.L. (2000) The ZIP family of metal transporters. Biochim. Biophys. Acta, 1465, 190–198. 10748254 10.1016/s0005-2736(00)00138-3

[pbi12795-bib-0017] Hall, S.M. and Baker, D.A. (1972) The chemical composition of Ricinus phloem exudate. Planta, 106, 131–140. 24477954 10.1007/BF00383992

[pbi12795-bib-0018] Hanikenne, M. , Talke, I.N. , Haydon, M.J. , Lanz, C. , Nolte, A. , Motte, P. , Kroymann, J. *et al*. (2008) Evolution of metal hyperaccumulation required *cis*‐regulatory changes and triplication of HMA4. Nature, 453, 391–395. 18425111 10.1038/nature06877

[pbi12795-bib-0019] He, J.L. , Li, H. , Luo, J. , Ma, C.F. , Li, S.J. , Qu, L. , Gai, Y. *et al*. (2013) A transcriptomic network underlies microstructural and physiological responses to cadmium in *Populus* x *canescens* . Plant Physiol. 162, 424–439. 23530184 10.1104/pp.113.215681 PMC3641221

[pbi12795-bib-0020] Jia, W.T. , Lv, S.L. , Feng, J.J. , Li, J.H. , Li, Y.X. and Li, S.Z. (2016) Morphophysiological characteristic analysis demonstrated the potential of sweet sorghum (*Sorghum bicolor* (L.) Moench) in the phytoremediation of cadmium‐contaminated soils. Environ. Sci. Pollut. Res. Int. 23, 18823–18831. 27318481 10.1007/s11356-016-7083-5

[pbi12795-bib-0021] Juwarkar, A.A. , Yadav, S.K. , Kumar, P. and Singh, S.K. (2008) Effect of biosludge and biofertilizer amendment on growth of *Jatropha curcas* in heavy metal contaminated soils. Environ. Monit. Assess. 145, 7–15. 17973198 10.1007/s10661-007-0012-9

[pbi12795-bib-0022] Kanehisa, M. and Goto, S. (2000) KEGG: kyoto encyclopedia of genes and genomes. Nucleic Acids Res. 28, 27–30. 10592173 10.1093/nar/28.1.27 PMC102409

[pbi12795-bib-0023] Kato, M. , Ishikawa, S. , Inagaki, K. , Chiba, K. , Hayashi, H. , Yanagisawa, S. and Yoneyama, T. (2010) Possible chemical forms of cadmium and varietal differences in cadmium concentrations in the phloem sap of rice plants (*Oryza sativa* L.). Soil Sci. Plant Nutr. 56, 839–847.

[pbi12795-bib-0024] Krämer, U. (2005) Phytoremediation: novel approaches to cleaning up polluted soils. Curr. Opin. Biotechnol. 16, 133–141. 15831377 10.1016/j.copbio.2005.02.006

[pbi12795-bib-0025] Lanquar, V. , Lelièvre, F. , Barbier‐Brygoo, H. and Thomine, S. (2004) Regulation and function of AtNRAMP4 metal transporter protein. Soil Sci. Plant Nutr. 50, 1141–1150.

[pbi12795-bib-0026] Lanquar, V. , Lelièvre, F. , Bolte, S. , Hamès, C. , Alcon, C. , Neumann, D. , Vansuyt, G. *et al*. (2005) Mobilization of vacuolar iron by AtNRAMP3 and AtNRAMP4 is essential for seed germination on low iron. EMBO J. 24, 4041–4051. 16270029 10.1038/sj.emboj.7600864 PMC1356305

[pbi12795-bib-0027] Lanquar, V. , Ramos, M.S. , Lelièvre, F. , Barbier‐Brygoo, H. , Krieger‐Liszkay, A. , Krämer, U. and Thomine, S. (2010) Export of vacuolar manganese by AtNRAMP3 and AtNRAMP4 is required for optimal photosynthesis and growth under manganese deficiency. Plant Physiol. 152, 1986–1999. 20181755 10.1104/pp.109.150946 PMC2850043

[pbi12795-bib-0028] Li, S.Z. (2013) The roadmap of the development of biofuel industry. China Brew. 32, 77–81.

[pbi12795-bib-0029] Lombi, E. , Tearall, K.L. , Howarth, J.R. , Zhao, F.J. , Hawkesford, M.J. and McGrath, S.P. (2002) Influence of iron status on cadmium and zinc uptake by different ecotypes of the hyperaccumulator *Thlaspi caerulescens* . Plant Physiol. 128, 1359–1367. 11950984 10.1104/pp.010731 PMC154263

[pbi12795-bib-0030] Lu, L.L. , Tian, S.K. , Yang, X.E. , Li, T.Q. and He, Z.L. (2009) Cadmium uptake and xylem loading are active processes in the hyperaccumulator *Sedum alfredii* . J. Plant Physiol. 166, 579–587. 18937997 10.1016/j.jplph.2008.09.001

[pbi12795-bib-0031] Lu, L.L. , Tian, S.K. , Zhang, J. , Yang, X.E. , Labavitch, J.M. , Webb, S.M. , Latimer, M. *et al*. (2013) Efficient xylem transport and phloem remobilization of Zn in the hyperaccumulator plant species *Sedum alfredii* . New Phytol. 198, 721–731. 23421478 10.1111/nph.12168

[pbi12795-bib-0032] Lux, A. , Martinka, M. , Vaculík, M. and White, P.J. (2011) Root responses to cadmium in the rhizosphere: a review. J. Exp. Bot. 62, 21–37. 20855455 10.1093/jxb/erq281

[pbi12795-bib-0033] Marchiol, L. , Fellet, G. , Perosa, D. and Zerbi, G. (2007) Removal of trace metals by *Sorghum bicolor* and *Helianthus annuus* in a site polluted by industrial wastes: a field experience. Plant Physiol. Biochem. 45, 379–387. 17507235 10.1016/j.plaphy.2007.03.018

[pbi12795-bib-0034] Martinka, M. and Lux, A. (2004) Response of roots of three populations of *Silene dioica* to cadmium treatment. Biologia, 59, 185–189.

[pbi12795-bib-0035] McGrath, S.P. , Lombi, E. , Gray, C.W. , Caille, N. , Dunham, S.J. and Zhao, F.J. (2006) Field evaluation of Cd and Zn phytoextraction potential by the hyperaccumulators *Thlaspi caerulescens* and *Arabidopsis halleri* . Environ. Pollut. 141, 115–125. 16202493 10.1016/j.envpol.2005.08.022

[pbi12795-bib-0036] Metwali, E.R. , Gowayed, S.H. , Al‐Maghrabi, O. and Mosleh, Y. (2013) Evaluation of toxic effect of copper and cadmium on growth, physiological traits and protein profile of wheat (*Triticum aestivium* L.), maize (*Zea mays* L.) and sorghum (*Sorghum bicolor* L.). World Appl. Sci. J. 21, 301–314.

[pbi12795-bib-0037] Meyer, C.J. , Seago, J.L. and Peterson, C.A. (2009) Environmental effects on the maturation of the endodermis and multiseriate exodermis of *Iris germanica* roots. Ann. Bot. 103, 687–702. 19151041 10.1093/aob/mcn255 PMC2707867

[pbi12795-bib-0038] Mills, R.F. , Krijger, G.C. , Baccarini, P.J. , Hall, J.L. and Williams, L.E. (2003) Functional expression of AtHMA4, a P_1B_‐type ATPase of the Zn/Co/Cd/Pb subclass. Plant J. 35, 164–176. 12848823 10.1046/j.1365-313x.2003.01790.x

[pbi12795-bib-0039] Nevo, Y. and Nelson, N. (2006) The NRAMP family of metal‐ion transporters. Biochim. Biophys. Acta, 1763, 609–620. 16908340 10.1016/j.bbamcr.2006.05.007

[pbi12795-bib-0040] Papoyan, A. and Kochian, L.V. (2004) Identification of *Thlaspi caerulescens* genes that may be involved in heavy metal hyperaccumulation and tolerance. Characterization of a novel heavy metal transporting ATPase. Plant Physiol. 136, 3814–3823. 15516513 10.1104/pp.104.044503 PMC527178

[pbi12795-bib-0041] Pence, N.S. , Larsen, P.B. , Ebbs, S.D. , Letham, D.L.D. , Lasat, M.M. , Garvin, D.F. , Eide, D. *et al*. (2000) The molecular physiology of heavy metal transport in the Zn/Cd hyperaccumulator *Thlaspi caerulescens* . Proc. Natl Acad. Sci. USA, 97, 4956–4960. 10781104 10.1073/pnas.97.9.4956 PMC18339

[pbi12795-bib-0042] Perfus‐Barbeoch, L. , Leonhardt, N. , Vavasseur, A. and Forestier, C. (2002) Heavy metal toxicity: cadmium permeates through calcium channels and disturbs the plant water status. Plant J. 32, 539–548. 12445125 10.1046/j.1365-313x.2002.01442.x

[pbi12795-bib-0043] Peterson, C.A. and Enstone, D.E. (1996) Functions of passage cells in the endodermis and exodermis of roots. Physiol. Plant. 97, 592–598.

[pbi12795-bib-0044] Plaza, S. , Tearall, K.L. , Zhao, F.J. , Buchner, P. , McGrath, S.P. and Hawkesford, M.J. (2007) Expression and functional analysis of metal transporter genes in two contrasting ecotypes of the hyperaccumulator *Thlaspi caerulescens* . J. Exp. Bot. 58, 1717–1728. 17404382 10.1093/jxb/erm025

[pbi12795-bib-0045] Redjala, T. , Zelko, I. , Sterckeman, T. , Legué, V. and Lux, A. (2011) Relationship between root structure and root cadmium uptake in maize. Environ. Exp. Bot. 71, 241–248.

[pbi12795-bib-0046] Rodriguez‐Celma, J. , Ceballos‐Laita, L. , Grusak, M.A. , Abadia, J. and Lopez‐Millan, A.F. (2016) Plant fluid proteomics: delving into the xylem sap, phloem sap and apoplastic fluid proteomes. Biochim. Biophys. Acta, 1864, 991–1002. 27033031 10.1016/j.bbapap.2016.03.014

[pbi12795-bib-0047] Sasaki, A. , Yamaji, N. , Yokosho, K. and Ma, J.F. (2012) Nramp5 is a major transporter responsible for manganese and cadmium uptake in rice. Plant Cell, 24, 2155–2167. 22589467 10.1105/tpc.112.096925 PMC3442593

[pbi12795-bib-0048] Schreiber, L. (2010) Transport barriers made of cutin, suberin and associated waxes. Trends Plant Sci. 15, 546–553. 20655799 10.1016/j.tplants.2010.06.004

[pbi12795-bib-0049] Schreiber, L. , Hartmann, K. , Skrabs, M. and Zeier, J. (1999) Apoplastic barriers in roots: chemical composition of endodermal and hypodermal cell walls. J. Exp. Bot. 50, 1267–1280.

[pbi12795-bib-0050] Singer, A.C. , Bell, T. , Heywood, C.A. , Smith, J.A. and Thompson, I.P. (2007) Phytoremediation of mixed‐contaminated soil using the hyperaccumulator plant *Alyssum lesbiacum*: evidence of histidine as a measure of phytoextractable nickel. Environ. Pollut. 147, 74–82. 17084494 10.1016/j.envpol.2006.08.029

[pbi12795-bib-0051] Smith, J.A. and Milburn, J.A. (1980) Osmoregulation and the control of phloem‐sap composition in *Ricinus communis* L. Planta, 148, 28–34. 24311262 10.1007/BF00385438

[pbi12795-bib-0052] Song, X. , Hu, X. , Ji, P. , Li, Y. , Chi, G. and Song, Y. (2012) Phytoremediation of cadmium‐contaminated farmland soil by the hyperaccumulator *Beta vulgaris* L. *var. cicla* . Bull. Environ. Contam. Toxicol. 88, 623–626. 22286610 10.1007/s00128-012-0524-z

[pbi12795-bib-0053] Soudek, P. , Petrová, Š. , Vaňková, R. , Song, J. and Vaněk, T. (2014) Accumulation of heavy metals using Sorghum sp. Chemosphere, 104, 15–24. 24268752 10.1016/j.chemosphere.2013.09.079

[pbi12795-bib-0054] Sun, J. , Cui, J. , Luo, C. , Gao, L. , Chen, Y. and Shen, Z. (2013) Contribution of cell walls, nonprotein thiols, and organic acids to cadmium resistance in two cabbage varieties. Arch. Environ. Contam. Toxicol. 64, 243–252. 23111495 10.1007/s00244-012-9824-x

[pbi12795-bib-0055] Thomine, S. , Lelièvre, F. , Debarbieux, E. , Schroeder, J.I. and Barbier‐Brygoo, H. (2003) AtNRAMP3, a multispecific vacuolar metal transporter involved in plant responses to iron deficiency. Plant J. 34, 685–695. 12787249 10.1046/j.1365-313x.2003.01760.x

[pbi12795-bib-0056] Tiwari, M. , Sharma, D. , Dwivedi, S. , Singh, M. , Tripathi, R.D. and Trivedi, P.K. (2014) Expression in Arabidopsis and cellular localization reveal involvement of rice NRAMP, OsNRAMP1, in arsenic transport and tolerance. Plant, Cell Environ. 37, 140–152. 23700971 10.1111/pce.12138

[pbi12795-bib-0057] Trapnell, C. , Williams, B.A. , Pertea, G. , Mortazavi, A. , Kwan, G. , van Baren, M.J. , Salzberg, S.L. *et al*. (2010) Transcript assembly and quantification by RNA‐Seq reveals unannotated transcripts and isoform switching during cell differentiation. Nat. Biotechnol. 28, 511–515. 20436464 10.1038/nbt.1621 PMC3146043

[pbi12795-bib-0058] Ueno, D. , Iwashita, T. , Zhao, F.J. and Ma, J.F. (2008) Characterization of Cd translocation and identification of the Cd form in xylem sap of the Cd‐hyperaccumulator *Arabidopsis halleri* . Plant Cell Physiol. 49, 540–548. 18281325 10.1093/pcp/pcn026

[pbi12795-bib-0059] Uraguchi, S. and Fujiwara, T. (2013) Rice breaks ground for cadmium‐free cereals. Curr. Opin. Plant Biol. 16, 328–334. 23587938 10.1016/j.pbi.2013.03.012

[pbi12795-bib-0060] Uraguchi, S. , Kamiya, T. , Sakamoto, T. , Kasai, K. , Sato, Y. , Nagamura, Y. , Yoshida, A. *et al*. (2011) Low‐affinity cation transporter (OsLCT1) regulates cadmium transport into rice grains. Proc. Natl Acad. Sci. USA, 108, 20959–20964. 22160725 10.1073/pnas.1116531109 PMC3248505

[pbi12795-bib-0061] Vaculík, M. , Lux, A. , Luxová, M. , Tanimoto, E. and Lichtscheidl, I. (2009) Silicon mitigates cadmium inhibitory effects in young maize plants. Environ. Exp. Bot. 67, 52–58.

[pbi12795-bib-0062] Verbruggen, N. , Hermans, C. and Schat, H. (2009) Molecular mechanisms of metal hyperaccumulation in plants. New Phytol. 181, 759–776. 19192189 10.1111/j.1469-8137.2008.02748.x

[pbi12795-bib-0063] Verret, F. , Gravot, A. , Auroy, P. , Leonhardt, N. , David, P. , Nussaume, L. , Vavasseur, A. *et al*. (2004) Overexpression of *AtHMA4* enhances root‐to‐shoot translocation of zinc and cadmium and plant metal tolerance. FEBS Lett. 576, 306–312. 15498553 10.1016/j.febslet.2004.09.023

[pbi12795-bib-0064] Vogt, T. (2010) Phenylpropanoid biosynthesis. Mol. Plant, 3, 2–20. 20035037 10.1093/mp/ssp106

[pbi12795-bib-0065] Wan, L. and Zhang, H. (2012) Cadmium toxicity: effects on cytoskeleton, vesicular trafficking and cell wall construction. Plant Signal. Behav. 7, 345–348. 22499203 10.4161/psb.18992 PMC3443916

[pbi12795-bib-0066] Williams, L.E. and Mills, R.F. (2005) P_1B_‐ATPases ‐ an ancient family of transition metal pumps with diverse functions in plants. Trends Plant Sci. 10, 491–502. 16154798 10.1016/j.tplants.2005.08.008

[pbi12795-bib-0067] Woods, J. (2001) The potential for energy production using sweet sorghum in southern Africa. Energy Sustain. Dev. 5, 31–38.

[pbi12795-bib-0068] Wu, Z. , Zhao, X. , Sun, X. , Tan, Q. , Tang, Y. , Nie, Z. and Hu, C. (2015) Xylem transport and gene expression play decisive roles in cadmium accumulation in shoots of two oilseed rape cultivars (*Brassica napus*). Chemosphere, 119, 1217–1223. 25460764 10.1016/j.chemosphere.2014.09.099

[pbi12795-bib-0069] Wu, D. , Yamaji, N. , Yamane, M. , Kashino‐Fujii, M. , Sato, K. and Ma, J.F. (2016) The HvNramp5 transporter mediates uptake of cadmium and manganese, but not iron. Plant Physiol. 172, 1899–1910. 27621428 10.1104/pp.16.01189 PMC5100758

[pbi12795-bib-0070] Xu, J. , Sun, J.H. , Du, L.G. and Liu, X.J. (2012) Comparative transcriptome analysis of cadmium responses in *Solanum nigrum* and *Solanum torvum* . New Phytol. 196, 110–124. 22809404 10.1111/j.1469-8137.2012.04235.x

[pbi12795-bib-0071] Xue, D.W. , Jiang, H. , Deng, X.X. , Zhang, X.Q. , Wang, H. , Xu, X.B. , Hu, J. *et al*. (2014) Comparative proteomic analysis provides new insights into cadmium accumulation in rice grain under cadmium stress. J. Hazard. Mater. 280, 269–278. 25164389 10.1016/j.jhazmat.2014.08.010

[pbi12795-bib-0072] Yan, J. , Wang, P. , Wang, P. , Yang, M. , Lian, X. , Tang, Z. , Huang, C.F. *et al*. (2016) A loss‐of‐function allele of OsHMA3 associated with high cadmium accumulation in shoots and grain of *Japonica* rice cultivars. Plant, Cell Environ. 39, 1941–1954. 27038090 10.1111/pce.12747

[pbi12795-bib-0073] Ye, W.L. , Wood, B.A. , Stroud, J.L. , Andralojc, P.J. , Raab, A. , McGrath, S.P. , Feldmann, J. *et al*. (2010) Arsenic speciation in phloem and xylem exudates of castor bean. Plant Physiol. 154, 1505–1513. 20870777 10.1104/pp.110.163261 PMC2971624

[pbi12795-bib-0074] Yu, J.L. , Zhang, T. , Zhong, J. , Zhang, X. and Tan, T.W. (2012) Biorefinery of sweet sorghum stem. Biotechnol. Adv. 30, 811–816. 22306167 10.1016/j.biotechadv.2012.01.014

[pbi12795-bib-0075] Zelko, I. and Lux, A. (2004) Effect of cadmium on Karwinskia humboldtiana roots. Biologia, 59, 205–209.

[pbi12795-bib-0076] Zhao, F.J. , Hamon, R.E. , Lombi, E. , McLaughlin, M.J. and McGrath, S.P. (2002) Characteristics of cadmium uptake in two contrasting ecotypes of the hyperaccumulator *Thlaspi caerulescens* . J. Exp. Bot. 53, 535–543. 11847252 10.1093/jexbot/53.368.535

[pbi12795-bib-0077] Zhuang, P. , Shu, W. , Li, Z. , Liao, B. , Li, J. and Shao, J. (2009) Removal of metals by sorghum plants from contaminated land. J. Exp. Sci. 21, 1432–1437. 10.1016/s1001-0742(08)62436-5 19999999

