# Supplementary figures and images for: Comparative transcriptome combined with morpho‐physiological analyses revealed key factors for differential cadmium accumulation in two contrasting sweet sorghum genotypes
Source: Plant Biotechnol J. 2017 Aug 3;16(2):558–71. doi: 10.1111/pbi.12795 (PMC5787832; doi:10.1111/pbi.12795)

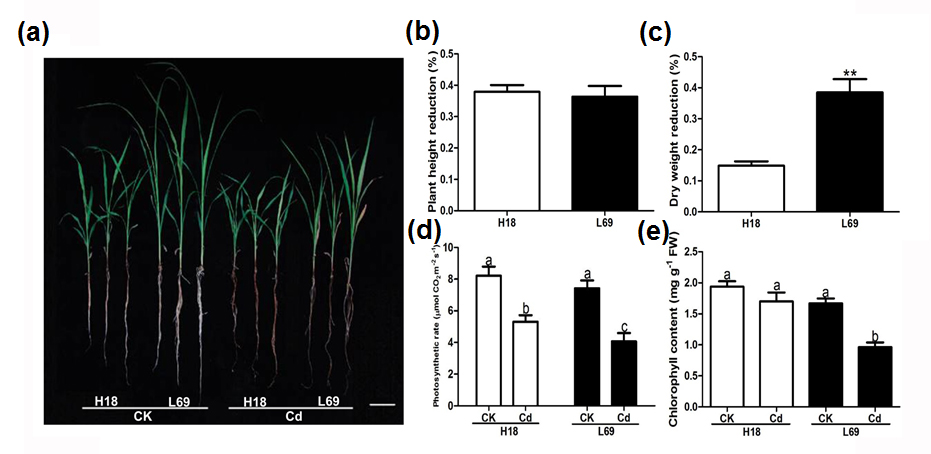

Supplement: Supplementary file 1 — Figure S1 Effects of cadmium on the growth of H18 and L69 seedlings. [file PBI-16-558-s014.jpg]

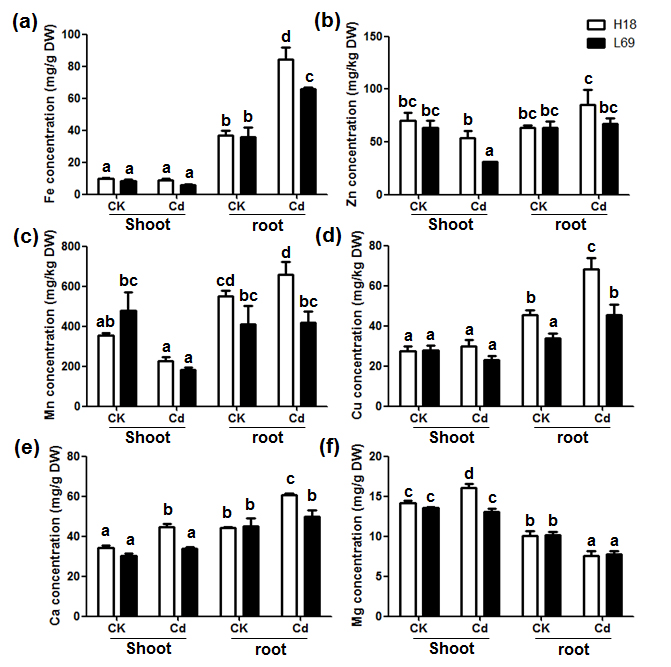

Supplement: Supplementary file 2 — Figure S2 Effects of Cd treatment on micronutrients accumulation. [file PBI-16-558-s013.jpg]

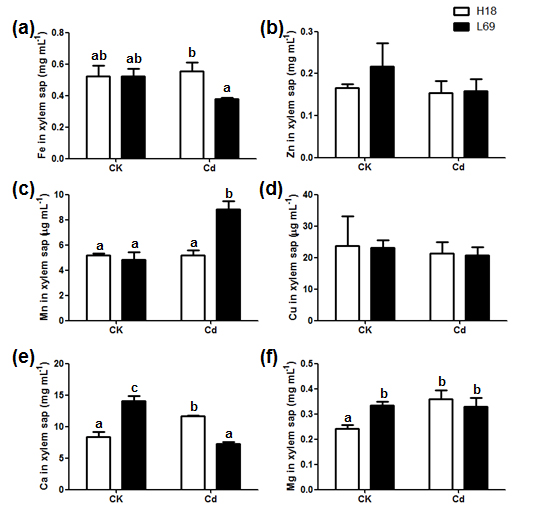

Supplement: Supplementary file 3 — Figure S3 Effects of Cd treatment on micronutrients contents in xylem sap. [file PBI-16-558-s012.jpg]

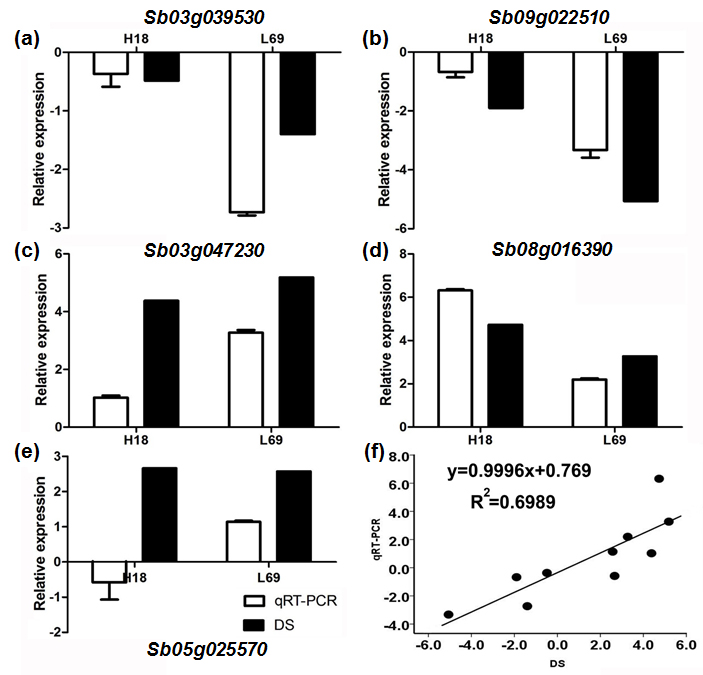

Supplement: Supplementary file 4 — Figure S4 qRT‐PCR validation of expression profiles of DEGs. [file PBI-16-558-s016.jpg]

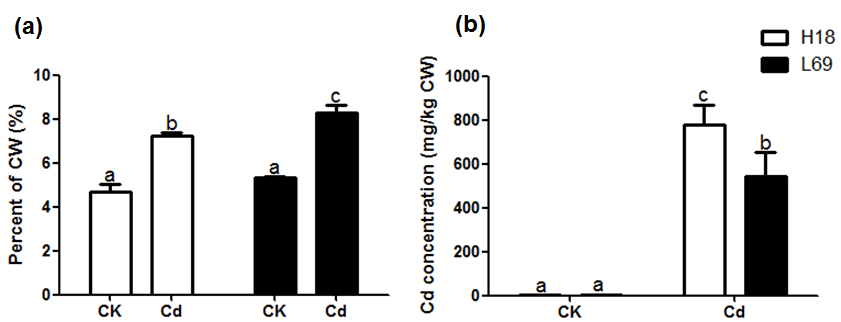

Supplement: Supplementary file 5 — Figure S5 The effect of Cd treatment on cell wall yield (a) and Cd concentration in CW (b) in H18 and L69 roots. [file PBI-16-558-s017.jpg]
