# Supplementary material for: Comparative transcriptome combined with morpho‐physiological analyses revealed key factors for differential cadmium accumulation in two contrasting sweet sorghum genotypes
Source: Plant Biotechnol J. 2017 Aug 3;16(2):558–71. doi: 10.1111/pbi.12795 (PMC5787832; doi:10.1111/pbi.12795)
Supplement: Supplementary file 6 — Table S1 The total protein concentrations and sugar composition in xylem exudates of H18 and L69 plants. [file PBI-16-558-s001.docx]

**Table S1. The total protein concentrations and sugar composition in xylem exudates of H18 and L69 plants** (values are means ± SE, n=3)**.**

| **Exudate** | **Total protein (µg/mL)** | **Sucrose (mM)** | **Glucose (mM)** | **Fructose (mM)** |
| --- | --- | --- | --- | --- |
| H18-CK | 10.04±1.37 | 0±0 | 0.13±0.03 | 0.12±0.02 |
| H18-Cd | 13.79±0.75 | 0.15±0.05 | 0.30±0.14 | 0.28±0.13 |
| L69-CK | 12.79±0.64 | 0±0 | 0.08±0.08 | 0.08±0.08 |
| L69-Cd | 14.33±0.80 | 0.20±0.08 | 0.12±0.03 | 0.10±0.002 |
