# Supplementary material for: Comparative transcriptome combined with morpho‐physiological analyses revealed key factors for differential cadmium accumulation in two contrasting sweet sorghum genotypes
Source: Plant Biotechnol J. 2017 Aug 3;16(2):558–71. doi: 10.1111/pbi.12795 (PMC5787832; doi:10.1111/pbi.12795)
Supplement: Supplementary file 17 — Appendix S1 Supplemental methods. [file PBI-16-558-s015.docx]

**Supplemental methods**

**Measurement of plant height and dry weight**

In hydroponic experiment, the plant height that is the distance from the bottom of shoot to the highest junction of sheath and leaf was measured after cadmium treatment for 14 days, and then the roots and shoots were harvested separately and dried at 70 °C until constant weight, which weighed as dry weight.

**Determination of photosynthetic rate and chlorophyll content**

The photosynthetic rate of the third leaf from the bottom was measured after cadmium treatment for 14 days using a portable photosynthetic system (LI-6400; Li-Cor Inc., Lincoln, NE, USA) with a light-emitting diode (LED) light source of 1000 μmol m^−2^ s^−1^. Meanwhile the third and fourth leaves from the bottom of caulicle were collected and weighed, then immersed in 95% ethanol for 3 days. Optical density (OD) of the extracts was measured at 665 and 649 nm for chlorophyll a and b, respectively (Lichtenthaler, 1987).

**Quantitative real-time PCR (qRT-PCR) analysis**

Two weeks-old hydroponically grown H18 and L69 seedlings were treated with 0 or 10 μM CdCl_2_ for 24 h, then roots were collected. Total RNA was isolated from harvested samples using Trizol reagent (TianGen, China), followed by purification with DNaseI treatment according to manufacturer’s instructions (Thermo Scientific Fermentas, USA). First strand cDNA was synthesised using a cDNA synthesis kit (Transgene, China). qRT-PCR was performed with the Stratagene MX3000P system (Agilent Stratagene, USA) using SYBR Green I (TOYOBO, Japan) to monitor double-stranded DNA synthesis. The *β-actin* gene was used as the internal control, and the relative expression levels of individual genes were calculated using the results from triplicate samples, as previously described ([Livak and Schmittgen, 2001](#_ENREF_2)). Three independent biological replicates were analysed, and similar results were obtained. The oligonucleotide primers used are listed in Table S11.

**Measurements of total protein and sugar composition in xylem sap**

Total protein concentrations were determined by the Bradford method with BSA as standard ([Bradford, 1976](#_ENREF_1)). The contents of glucose (Glc), fructose (Fru) and sucrose (Suc) in xylem sap were determined using the Suc/D-Glc/D-Fru assay kit (Megazyme, Ireland) following the manufacturer’s recommendations ([Lv et al., 2015](#_ENREF_3)).

**Cell wall extraction**

The extraction of cell wall was carried out as described by Zhu et al. (2012) with minor modifications. Roots of sweet sorghum seedlings treated with 0 and 10 μM CdCl_2_ for 14 days were grounded with a mortar and pestle in liquid nitrogen. Then the power was homogenized with 75% ethanol and kept in an ice-cold water bath for 2 h. The samples were centrifuged at 8000 g for 10 min and the supernatant was removed. Moreover the pellets were homogenized and washed with acetone, methanol: chloroform at a ratio of 1:1, and methanol for 20 min each. After each washing step, the samples were centrifuged at 8000 g for 2 min and the supernatant was removed. The final pellet of cell walls was dried at 70 °C for further use.

**References**

Bradford, M.M. (1976) A rapid and sensitive method for the quantitation of microgram quantities of protein utilizing the principle of protein-dye binding. *Anal. Biochem.* **72**, 248-254.

Lichtenthaler HK (1987) Chlorophylls and carotenoids: pigments of photosynthetic biomembranes. *Method Enzymol.* **18**, 350-382.

Livak, K.J. and Schmittgen, T.D. (2001) Analysis of relative gene expression data using real-time quantitative PCR and the 2(-^∆∆^C_T_) method. *Methods*, **25**, 402-408.

Lv, S., Jiang, P., Nie, L., Chen, X., Tai, F., Wang, D., Fan, P., Feng, J., Bao, H., Wang, J. and Li, Y. (2015) H(+) -pyrophosphatase from *Salicornia europaea* confers tolerance to simultaneously occurring salt stress and nitrogen deficiency in Arabidopsis and wheat. *Plant Cell Environ.* **38**, 2433-2449.

Zhu, X.F., Lei, G.J., Jiang, T., Liu, Y., Li, G.X. and Zheng, S.J. (2012) Cell wall polysaccharides are involved in P-deficiency-induced Cd exclusion in Arabidopsis thaliana. *Planta*, **236**, 989-997.
